# Sirtuin 1 alleviates neuroinflammation‐induced apoptosis after traumatic brain injury

**DOI:** 10.1111/jcmm.16534

**Published:** 2021-04-08

**Authors:** Guan Wei, Jiawei Wang, Yanbin Wu, Xiaoxin Zheng, Yile Zeng, Yasong Li, Xiangrong Chen

**Affiliations:** ^1^ Department of Emergency The Second Affiliated Hospital Fujian Medical University Quanzhou China; ^2^ Department of Neurosurgery Anxi County Hospital of Traditional Chinese Medicine Quanzhou China; ^3^ Department of Neurosurgery The Second Affiliated Hospital Fujian Medical University Quanzhou China

**Keywords:** neuroinflammation, neuronal apoptosis, NF‐κB, sirtuin 1, traumatic brain injury

## Abstract

Sirtuin 1 (SIRT1) plays a very important role in a wide range of biological responses, such as metabolism, inflammation and cell apoptosis. Changes in the levels of SIRT1 have been detected in the brain after traumatic brain injury (TBI). Further, SIRT1 has shown a neuroprotective effect in some models of neuronal death; however, its role and working mechanisms are not well understood in the model of TBI. This study aimed to address this issue. SIRT1‐specific inhibitor (sirtinol) and activator (A3) were introduced to explore the role of SIRT1 in cell apoptosis. Results of the study suggest that SIRT1 plays an important role in neuronal apoptosis after TBI by inhibiting NF‐κB, IL‐6 and TNF‐α deacetylation and the apoptotic pathway sequentially, possibly by alleviating neuroinflammation.

## 1 INTRODUCTION

The pathophysiological features of traumatic brain injury (TBI) include primary brain injuries, which are difficult to predict, and secondary brain injury.^1^, ^2^ Over the course of TBI, neuronal apoptosis occurs through a complex sequence of pathological events that involve several contributing factors, such as excitotoxicity, excitotoxic damage, mitochondrial dysfunction and autophagy.^3^ Among these events, inflammation is an important factor that aggravates nerve injury during the process of secondary insult. Although the exact mechanisms responsible for the occurrence of neuronal insult and their final degeneration are equivocal, vast literature suggests an important role of neuroinflammation.^4^, ^5^ Cytokine signalling and inflammasome are linked to the ‘redox‐sensitive’ master transcriptional regulator nuclear factor‐kappa B (NF‐κB). Indeed, activation of NF‐κB causes an increase in the interleukin (IL)‐1 level, which promotes the cascade of inflammatory factors in microglia (ie TNF‐α) and astrocytes (ie IL‐6).^6^, ^7^ Accordingly, restrained activation of NF‐κB reduces the neuroinflammatory process, which can prevent neuronal apoptosis.^8^

Sirtuin 1 (SIRT1), also known as nicotinamide adenine dinucleotide (NAD)‐dependent deacetylase sirtuin‐1, contributes to cellular regulation (reaction to stressors and longevity).^9^ It is a highly conserved and well‐characterized NAD‐dependent class III histone deacetylase in mammalian cells. Numerous studies have demonstrated that SIRT1 plays a central role in the regulation of different biological processes, such as mitochondrial biogenesis, oxidation and inflammation.^10^, ^11^, ^12^ SIRT1 is activated in many central nervous system diseases, such as subarachnoid haemorrhage and Parkinson's disease.^13^ In addition, it has been reported that it is an important regulator for protecting against TBI‐induced secondary brain injury.^14^, ^15^ It is reasonably believed that the activation of SIRT1 could be involved in the post‐TBI neuroprotective effect.^14^, ^16^ However, it is important to explore whether the protective effects of SIRT1 on cellular apoptosis after TBI were related to alleviation of neuroinflammation. Therefore, the present study aimed to examine the effect of SIRT1 on acute inflammatory responses and cellular apoptosis during the acute phase of TBI.

## 2 MATERIALS AND METHODS

### 2.1 Animals

Male Sprague‐Dawley rats (weight, 280‐300 g) were obtained from the Animal Center of Fujian Medical University, Quanzhou, China. The animals were maintained in controlled temperature (37.0 ± 0.5°C) conditions on a 12 hours/12 hours light/dark cycle. All rats were maintained on an ad libitum diet for 1 week before the experiment. All experimental protocols were approved by the Institutional Animal Care and Use Committee of Fujian Medical University and were in accordance with the guide of the Care and Use of Laboratory Animals by the National Institutes of Health.

### 2.2 TBI model

The rats were placed in a stereotaxic frame after being anaesthetized by exposure to isoflurane. A 2 cm midline scalp incision was made to expose the skull. Then, a 6 mm hole was made over the left parietal cortex, and the centre of the hole was 2.5 mm lateral to the midline on the mid‐coronal plane. The dura was not torn during the operation. Then, a 40 g weight was released onto the dura from 25 cm along a stainless steel string. The scalp wound was sutured, and the animals were placed back in their cages. Sham animals received the same procedures except that they were not injured by releasing the weight.

### 2.3 Experimental design

Part 1: A total of 33 rats were used in this experiment. Three rats died after the operation and were excluded. A total of 24 rats were divided into four groups post‐TBI, and rats in three groups were killed at 6, 12, 24, 48 and 72 hours after TBI (n = 6 each). Then, six rats in each group were subjected to Western blot analysis at peak SIRT1 expression (n = 6 each).

Part 2: Rats (112 rats were used, and 22 rats died) were divided into five groups (n = 18 each) to determine the role of SIRT1 after TBI: Sham group; TBI group; TBI + vehicle group; TBI + activator 3 (A3) group and TBI + sirtinol group. All animals were killed 24 hours post‐TBI after neurological function measurement. The rats were evaluated for neurological function and cerebral oedema, and they were subjected to Western blotting and a terminal deoxynucleotidyl transferase dUTP nick end labelling (TUNEL) assay.

Part 3: Seventy‐two rats (101 rats were used, and 11 rats died) were placed in the following groups (n = 18 each) to explore the relationship between SIRT1 and neuroinflammation: Sham group; TBI group; TBI + vehicle group; TBI + activator 3 (A3) group and TBI + sirtinol group. The animals were killed 24 hours post‐TBI for Western blot and TUNEL assay.

### 2.4 Drug administration

All drugs were dissolved in a vehicle (0.9% saline + 3% dimethyl sulphoxide) (Sigma, St. Louis, MO, USA). Sirtinol (2 mmol/L, 30 μL/kg) or vehicle was injected intraperitoneally 2 hours before TBI. A3 (Santa Cruz Biotechnology, Santa Cruz, CA, USA) was diluted to 5 mg/kg. The rats were intraperitoneally injected with A3 30 minutes after TBI. The drug dose was confirmed in a previous study.^17^

### 2.5 Western blot analysis

Protein extracts were separated by 10% or 15% sodium dodecyl sulphate‐polyacrylamide gel electrophoresis and transferred to polyvinylidene fluoride membranes (Bio‐Rad Laboratories, Hercules, CA, USA). The membranes were blocked with 5% non‐fat milk for 2 hours, and then they were incubated overnight at 4°C with SIRT1 (1:200, Santa Cruz Biotechnology), Bcl‐2 (1:200, Santa Cruz Biotechnology), Cleaved caspase‐3 (1:1000, Cell Signaling Technology, Danvers, MA, USA), Bax (1:200, Santa Cruz Biotechnology), caspase‐3 (1:400, Cell Signaling Technology), ac‐NF‐кB (1:400, Cell Signaling Technology), IL‐6 (1:200, Santa Cruz Biotechnology), TNF‐α (1:200, Santa Cruz Biotechnology) and β‐actin (1:5000, Bioworld Technology, St. Louis Park, MN, USA) in blocking buffer. After three washes with TBST for 15 minutes each, the membranes were subsequently incubated in secondary antibody conjugated with horseradish peroxidase (HRP) (1:1000, Bioworld Technology) at room temperature for 2 hours. The protein bands were exposed to the Tanon‐5200 Chemiluminescent Imaging System, and strip grey levels were quantified by software (version 4.62; Bio‐Rad Laboratories).

### 2.6 Immunohistochemical staining

Brain tissue samples were cut to 5 μm after fixation in formalin for 24 hours. The sections were incubated overnight at 4°C with primary antibody against SIRT1 (1:50, Santa Cruz Biotechnology). After three 15 minutes washes in PBS, the sections were incubated with HRP‐conjugated IgG (1:500; Bioworld Technology) for 60 minutes. After three washes for 30 minutes each, counterstaining was performed with diaminobenzidine and haematoxylin. Control tissue was not exposed to the primary antibody.

### 2.7 TUNEL analysis

Apoptotic cells were determined using a TUNEL detection kit (Roche Inc, Indianapolis, IN, USA). The sections were incubated at 37°C with TUNEL reaction fluid for 60 minutes. The slides were washed three times with PBS for 45 minutes, followed by blocking with 10% goat serum in 0.1 mol/L Tris for 15 minutes. The slides were covered with slide glass and mounting medium after three washes. DNA was fixed with streptavidin‐HRP peroxidase (1:40 dilution) and dyed with DAB Chromogen. The apoptotic cells were atrophied with a crimped brown nucleus according to the microscopic analysis (ZEISS HB050 inverted microscope; Carl Zeiss, Thornwood, NY, USA). The positive cells were monitored and analysed by two observers blinded to the experiment. TUNEL‐positive cells from each sample were selected for quantification. The average percentage of the four aspects was regarded as the data for analysis.

### 2.8 Brain water content

Animals were anaesthetized as described above. The brain stem and cerebellum were removed, and the left cerebral hemisphere was separated. The wet weight (ww) of fresh tissue was determined after removing the brain. The hemispheres were parched at 80°C for 72 hours, and dry weight (dw) was measured in succession. The percentage of brain water content was calculated by the following formula: ((ww – dw)/ww).

### 2.9 Neurobehavioural evaluation

Neurological deficit was evaluated 24 hours and 3 days after TBI using the beam‐walking test. The investigators performed seven different tasks to test motor function, balance and alertness. One point was given for failing on all tasks; 0 = minimum deficit and 7 = maximum deficit. The severity of injury was defined by the initial grade and evaluated 1 hour after TBI, and it is a reliable predictor of the final outcome. The behavioural test was carried out by two independent investigators who had no prior knowledge of the experimental groups.

### 2.10 Statistical analysis

All statistical analyses were performed with SPSS 17.0 software (SPSS Inc, Chicago, IL, USA). Data are expressed as mean with standard deviation, and the differences were analysed using analysis of variance and Tukey's test. A *P*‐value <.05 was considered significant.

## 3 RESULTS

### 3.1 Expression of SIRT1 in the brain cortex after TBI

**FIGURE 1:**
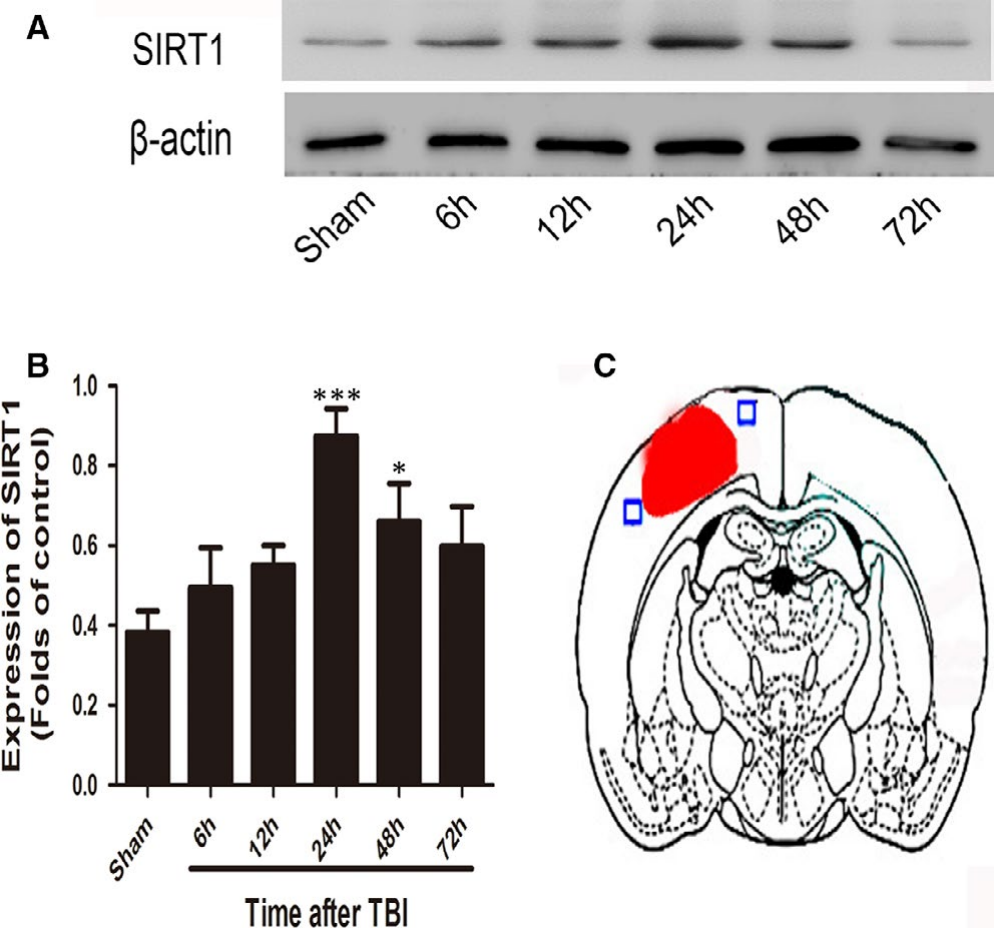
Expression of SIRT1 in the brain cortex after TBI. Western blot analysis shows the expression of SIRT1 at 6, 12, 24, 48 and 72 h after TBI; *P < .05, ***P < .001 vs. Sham group

Western blot was performed to evaluate the time course of SIRT1 expression after TBI. The SIRT1 level peaked at 24 hours and remained high until 48 hours following TBI (Figure 1). Significant variation was detected among the Sham, 24 hours and 48 hours groups.

### 3.2 Effects of sirtinol on SIRT1 expression and neuroinflammation under TBI conditions

**FIGURE 2:**
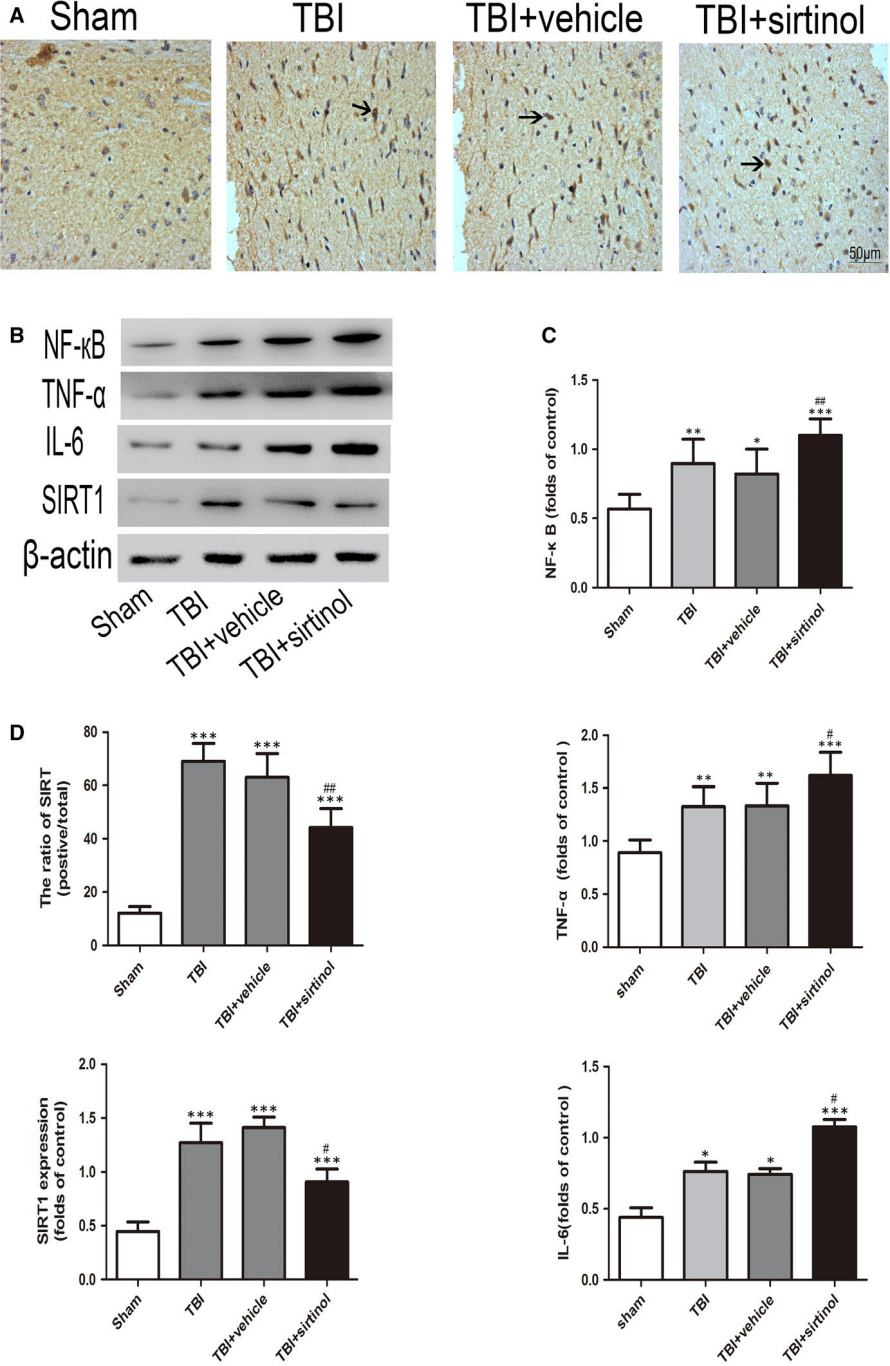
Effects of sirtinol on the expression of SIRT1 (A, B, D) and inflammatory factor (B, C) 24 h after TBI. Bars represent mean ± SD. *P < .05, **P < .01, ***P < .001 vs. Sham group; ^#^ *P* < .05, ^##^ *P* < .01 vs. TBI + vehicle group

As shown in Figure 2A, the expression of SIRT1 increased significantly after TBI compared with that in the sham group. On the contrary, sirtinol administration significantly reduced the level of SIRT1. Similarly, immunohistochemical staining (Figure 2B) revealed elevation of the percentage of SIRT1‐positive neurons in the cerebral cortex after TBI, which decreased after sirtinol administration.

To investigate the effect of sirtinol on SIRT1 expression, the levels of NF‐кB, IL‐6 and TNF‐α were evaluated by Western blot (Figure 2B,C). Compared with the sham group, the levels of NF‐кB, IL‐6 and TNF‐α were significantly increased after TBI. After sirtinol treatment, the NF‐кB, IL‐6 and TNF‐α expression levels were further promoted compared with those in the TBI + vehicle group.

### 3.3 Effects of sirtinol on brain oedema and neurological function

**FIGURE 3:**
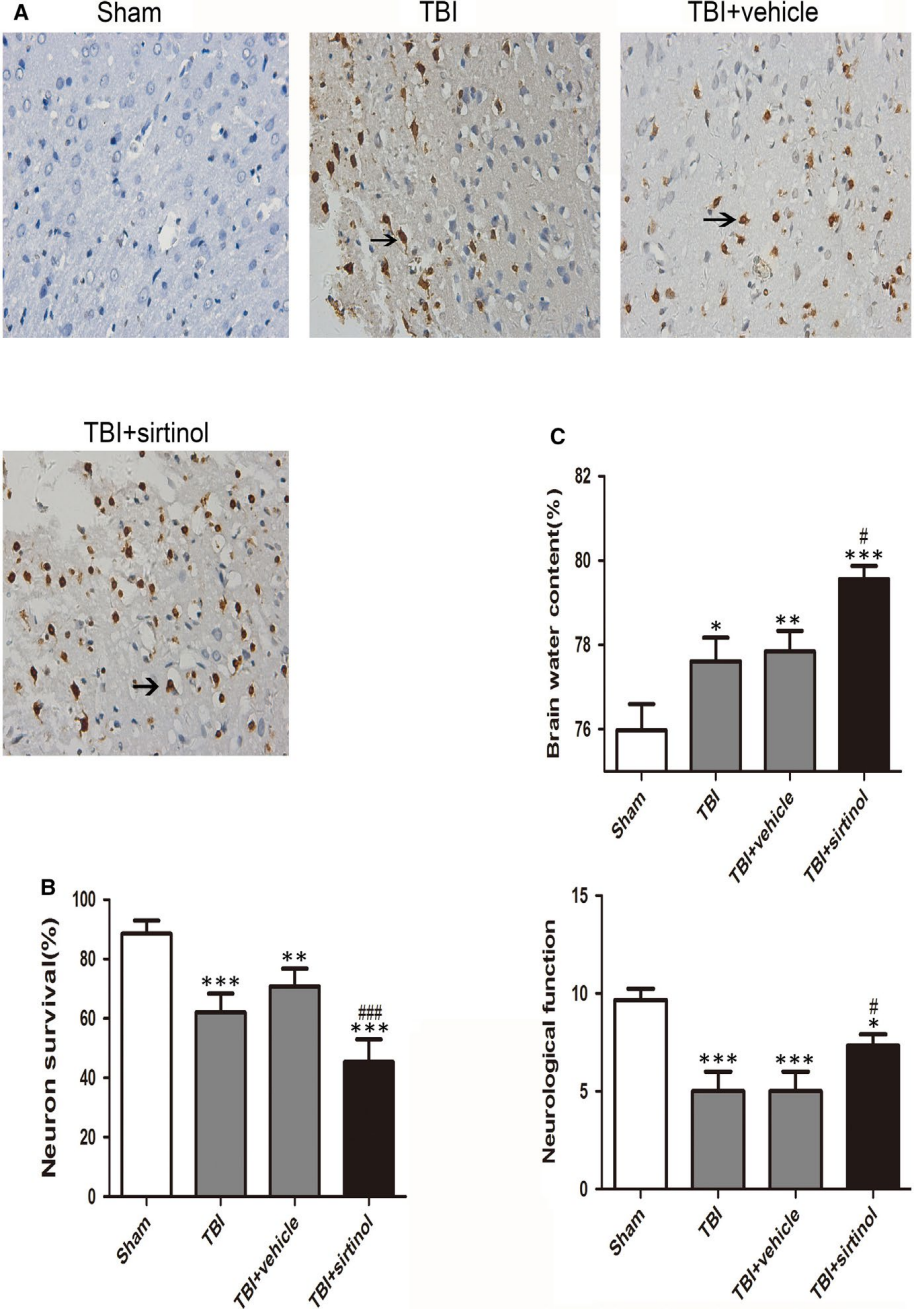
Effects of sirtinol on neuronal survival 24 h after TBI (A, B) The apoptotic index was determined by the TUNEL assay 1 d after TBI. (C) Brain water content and neurological function were assessed after TBI. Data represent mean ± SD, *P < .05, **P < .01, ***P < .001 vs. Sham group; ^#^ *P* < .05, ^###^ *P* < .001 vs. TBI + vehicle group

The brain water content was significantly increased after TBI (Figure 3C). Further, sirtinol restrained SIRT1 and further raised the water content of the cerebral cortex compared with that in the TBI + vehicle group.

After 24 hours of TBI, the scores in the TBI and TBI + vehicle groups were lower than that in the Sham group. After sirtinol administration, the neurological score was significantly promoted than that in the TBI + vehicle group (Figure 3C). The sirtinol‐treated group showed neurological deficits compared with the TBI + vehicle group.

### 3.4 Effects of sirtinol on neuronal apoptosis

TUNEL‐positive cells were detected, a few positive neurons in the rat brain, in the Sham group (Figure 3A,B). There were much more TUNEL‐positive neurons in the TBI and TBI + vehicle groups. However, the percentage of TUNEL‐positive neurons was raised after sirtinol treatment.

**FIGURE 4:**
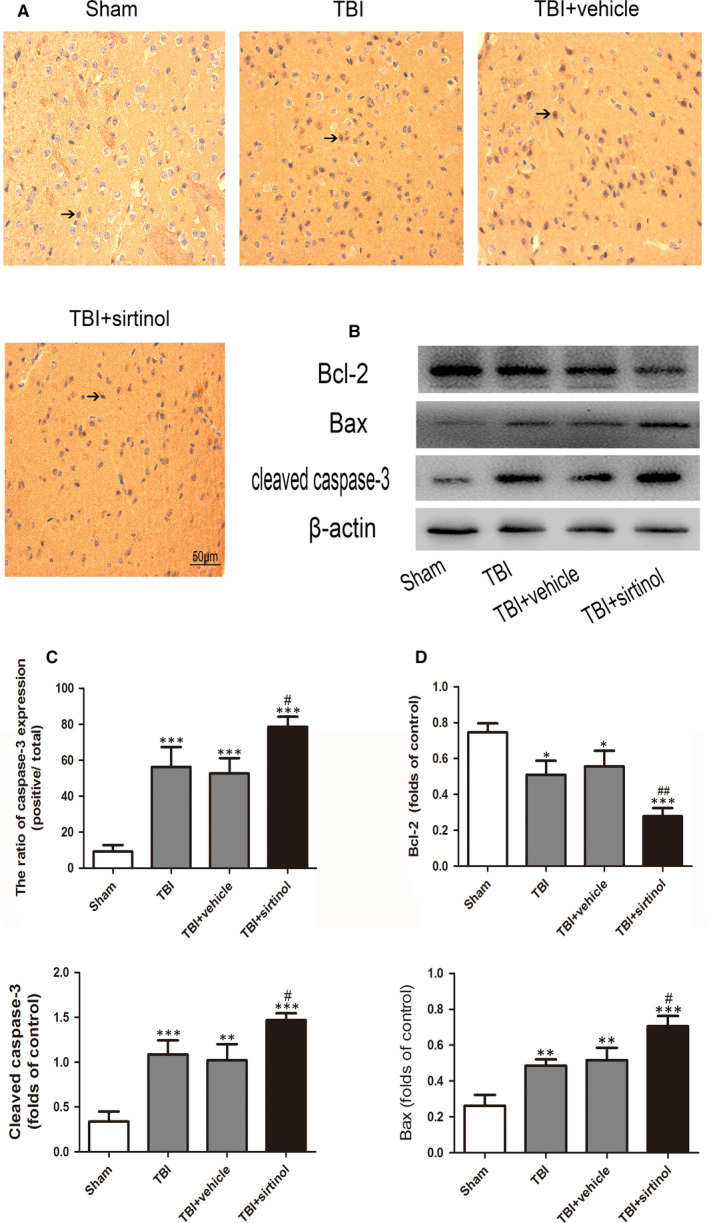
Effects of sirtinol on the SIRT1 and apoptotic protein expression after TBI. A, Representative photomicrographs of caspase‐3 staining in the experimental groups. B, Effects of sirtinol on the apoptotic pathway, including the levels of cleaved caspase‐3 Bax and Bcl‐2. Bars represent mean ± SD. *P < .05, **P < .01 and ***P < .001 vs. Sham group; ^#^ *P* < .05, ^##^ *P* < 0.001vs. TBI + vehicle group

Compared with the TBI + vehicle group, sirtinol administration significantly elevated cleaved caspase‐3 and Bax, and deduced the level of Bcl‐2. In addition, the percentage of caspase‐3‐positive neurons was significantly increased after sirtinol administration compared with that in the TBI + vehicle group (Figure 4).

### 3.5 Effects of A3 on the expression of SIRT 1, neuronal apoptosis, brain water content and neurological function

**FIGURE 5:**
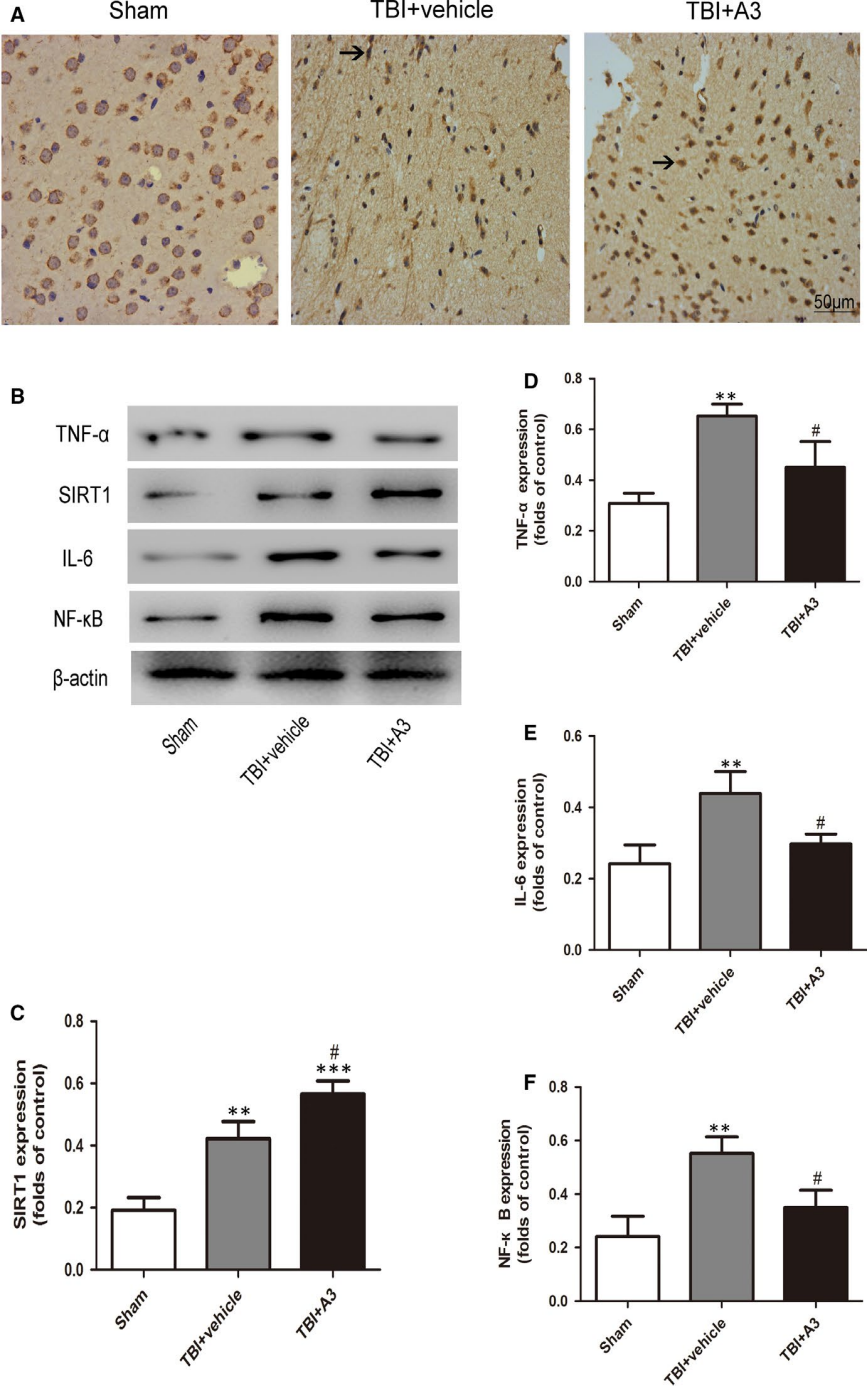
Effects of A3 on the SIRT1 expression and neuroinflammation 24 h after TBI. A, Representative Immunohistochemical staining to detect A3 on the expression of SIRT1. B, The levels of SIRT1, NF‐кB, TNF‐α and IL‐6 were significantly increased after TBI and were evidently decreased by the A3 treatment. Arrows point to SIRT1‐positive neurons. Data represent mean ± SD, **P < .01, ***P < .001 vs. Sham group; ^#^ *P* < .05 vs. TBI + vehicle group

**FIGURE 6:**
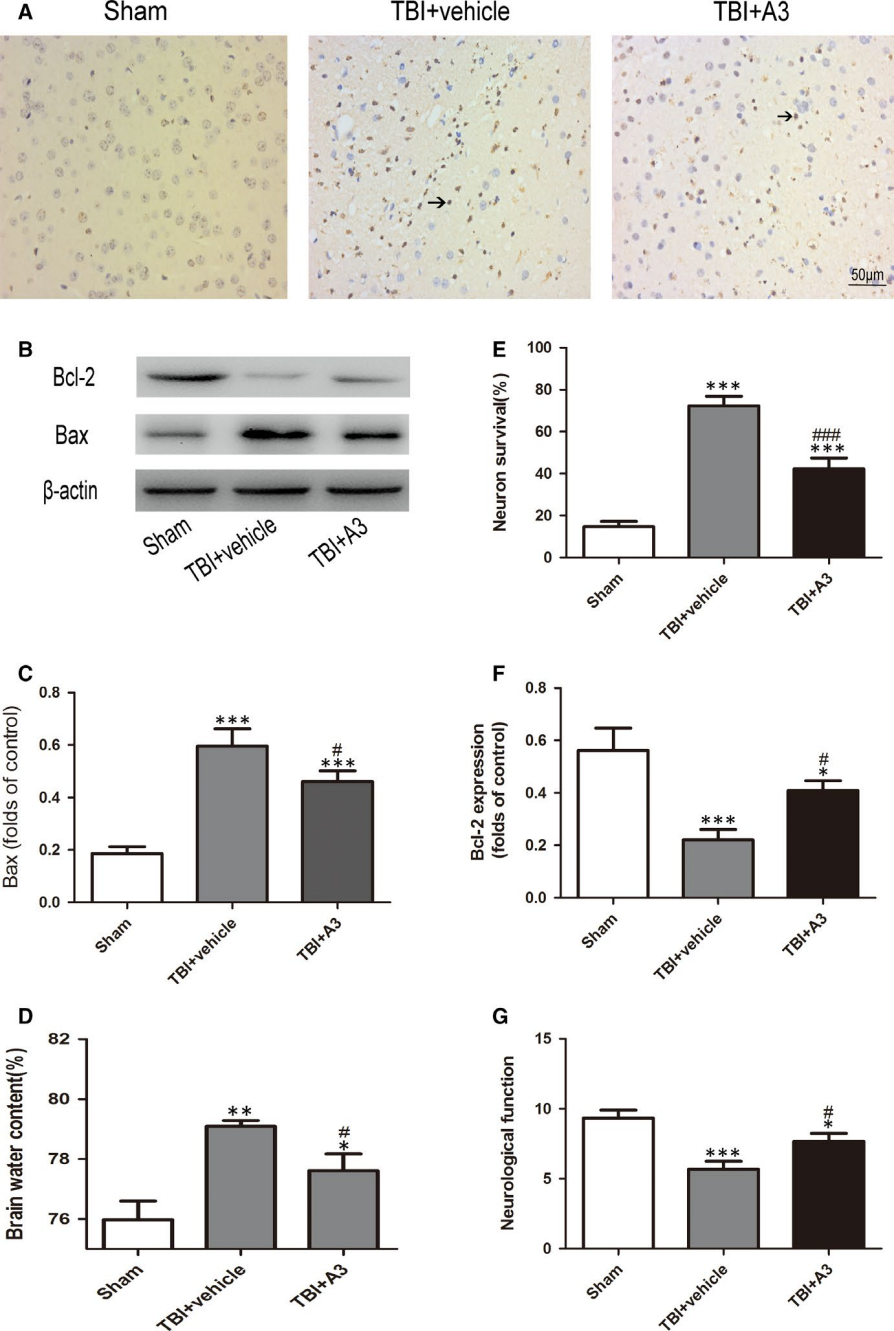
Effects of A3 on apoptosis, brain oedema and neurological function 24 h after TBI. (A, E) Representative photomicrographs of TUNEL staining in the experimental groups. (B, C, F) A3 treatment significantly decreased the Bax protein levels, but increased the Bcl‐2 protein levels compared with those in the TBI + vehicle group. (D, G) Impaired brain oedema and neurological behaviour decreased significantly after A3 administration, compared with those in the TBI + vehicle group. Data represent mean ± SD, *P < .05, **P < .01, ***P < .001 vs. Sham group; ^#^ *P* < .05, ^##^ *P* < .01 vs. TBI + vehicle group

As shown in Figure 5, Western blot and immunohistochemical staining revealed that A3 treatment significantly increased the level of SIRT1 compared with that in the TBI + vehicle group. Western blot presented that A3 administration could impair the Bax level and raise the level of Bcl‐2 (Figure 6). Further, neuronal apoptosis was reduced following A3 administration (Figure 6A). Results of brain oedema and neurological function showed that administration of A3 could markedly alleviate the damage of neurologic behaviour and brain oedema compared with that in the TBI + vehicle group (Figure 6D,G).

## 4 DISCUSSION

SIRT1 is a histone deacetylase that regulates signal transcription, cell apoptosis, oxidative stress and inflammation through deacetylation of intracellular signalling molecules.^18^ Previous studies have demonstrated that SIRT1 plays a key role in different central nervous system diseases.^19^, ^20^, ^21^ Overexpression or activation of SIRT1 may play a protective role in different types of nervous system injury, such as brain ischaemia and TBI.^16^, ^17^ In addition, SIRT1 has been found to affect neuronal plasticity, neuronal apoptosis and cognitive functioning.^22^, ^23^, ^24^ In this study, expression of SIRT1 was evaluated during the early period after TBI. SIRT1 expression was time‐dependently increased in the brain cortex, and it peaked 24 hours post‐TBI. Moreover, both cytosolic and nucleic SIRT1 expressions were enhanced significantly, suggesting that activation of SIRT1 in neural cells might play an important role post‐TBI.

It has been demonstrated that an inflammatory response was closely associated with neuronal apoptosis following TBI.^25^, ^26^ Activation of NF‐*κ*B mediates production of pro‐inflammatory cytokines, chemokines and inducible enzymes, namely which result in neuroinflammation.^27^ It would move to different tissue sites and cause tissue injury which leads to functional and structural changes. It is known that the degree of neuron apoptosis is related to TNF‐α messenger ribonucleic acid (mRNA) levels, and there is a possibility that a threshold for TNF‐α concentration is required for the initiation of apoptotic pathways.^28^ In the current study, large quantities of inflammatory cytokines (NF‐кB, IL‐6 and TNF‐α) were released after TBI, leading to significant neuronal apoptosis. Additionally, inhibition of SIRT1 with sirtinol compounded the damage to neurons caused by the secondary inflammatory response. These results were in accordance with previous studies demonstrating that SIRT1 could protect cells against various stresses, including inflammation and oxidative stress. In contrast, administration of A3 alleviated acetylation of NF‐кB, suggesting that SIRT1 may have a neuroprotective role after TBI by inhibiting the neuroinflammatory‐induced apoptotic pathways.

The potential roles of SIRT1 were further evaluated using a specific activator (A3). The results showed that suppression of SIRT1 aggravated neuronal apoptosis and brain oedema following TBI. To confirm whether activation of SIRT1 provided a neuroprotective effect, A3 was used in subsequent experiments. As expected, 5 mg/kg A3 efficiently ameliorated brain oedema, neuronal apoptosis and the neurofunction deficit after TBI. Taken together, these results strongly demonstrate a neuroprotective role of SIRT1 following TBI.

In this study, the protective actions of SIRT1 were exhibited by suppressing neuroinflammation after TBI. However, several limitations should be mentioned. Activated SIRT1 has numerous biological functions. First, this study lacked illumination of the role of SIRT1 in the long‐term recovery process. In addition, the relationship between neuroinflammation and other underlying mechanisms induced by SIRT1 warrants further study.

## CONFLICT OF INTEREST

All of the authors declare that there were no competing interests.

## AUTHOR CONTRIBUTIONS

**Guan Wei:** Writing‐original draft (equal). **Jiawei Wang:** Conceptualization (equal); Software (supporting). **Yanbin Wu:** Conceptualization (equal); Visualization (equal); Writing‐review & editing (equal). **Xiaoxin Zheng:** Investigation (equal); Writing‐review & editing (equal). **Yile Zeng:** Data curation (equal); Methodology (supporting); Software (equal). **Yasong Li:** Formal analysis (equal); Writing‐original draft (equal). **Xiangrong Chen:** Methodology (equal); Project administration (lead).

## Data Availability

The authors confirm that the data supporting the findings of this study are available within the article and its supplementary.
