# Corrigendum

**DOI:** 10.1111/jcmm.17548

**Published:** 2022-10-10

In Guan Wei et al.,^1^ the published article contains errors in Figures 4A and 6A. The correct figures are shown below. The authors confirm all results, and conclusions of this article remain unchanged.

**FIGURE 4:**
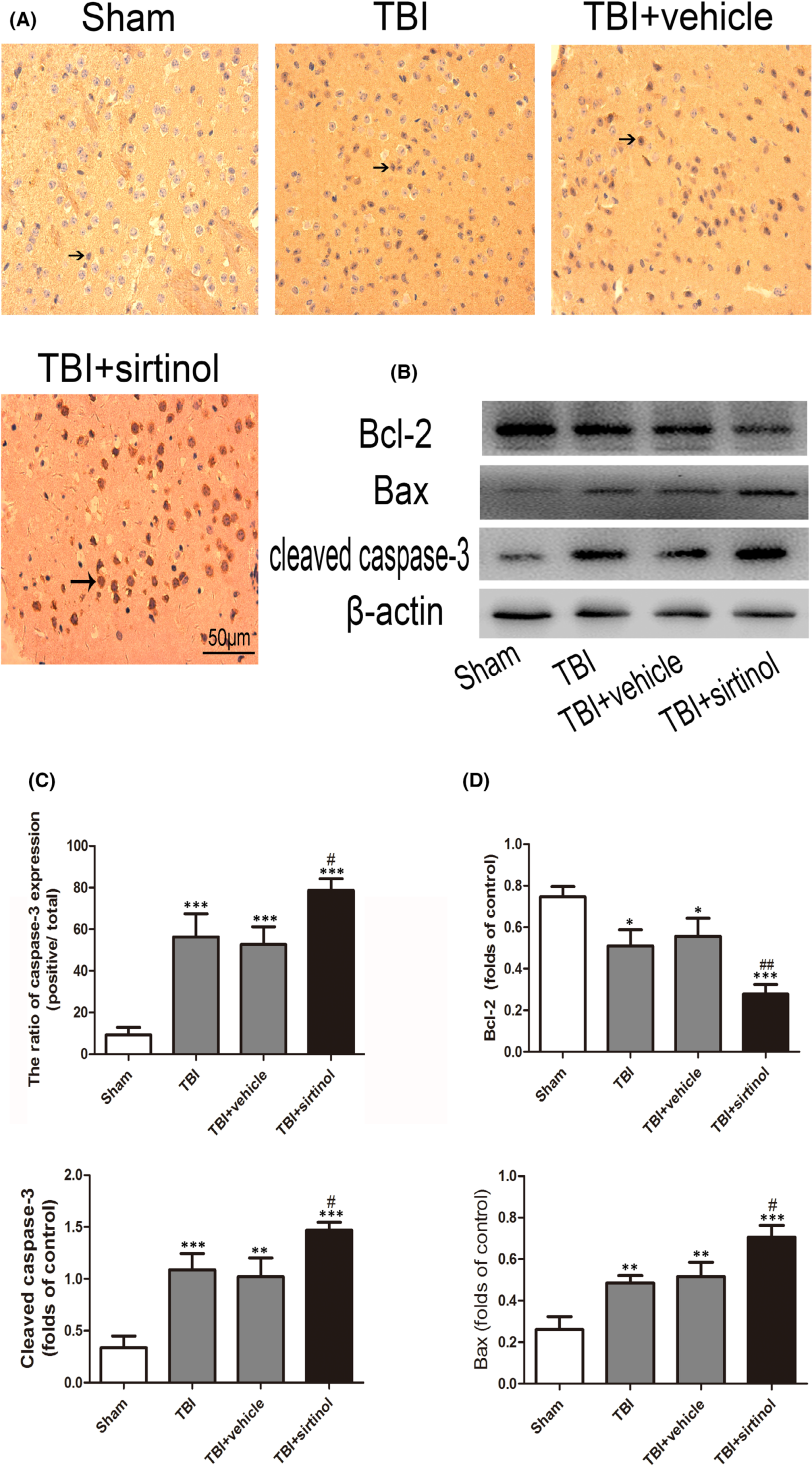
Effects of sirtinol on the SIRT1 and apoptotic protein expression after TBI. (A) Representative photomicrographs of caspase‐3 staining in the experimental groups. (B) Effects of sirtinol on the apoptotic pathway, including the levels of cleaved caspase‐3 Bax and Bcl‐2. Bars represent mean ± SD. *p < 0.05, **p < 0.01 and ***p < 0.001 versus Sham group; ^#^ *p* < 0.05, ^##^ *p* < 0.001 versus TBI + vehicle group

**FIGURE 6:**
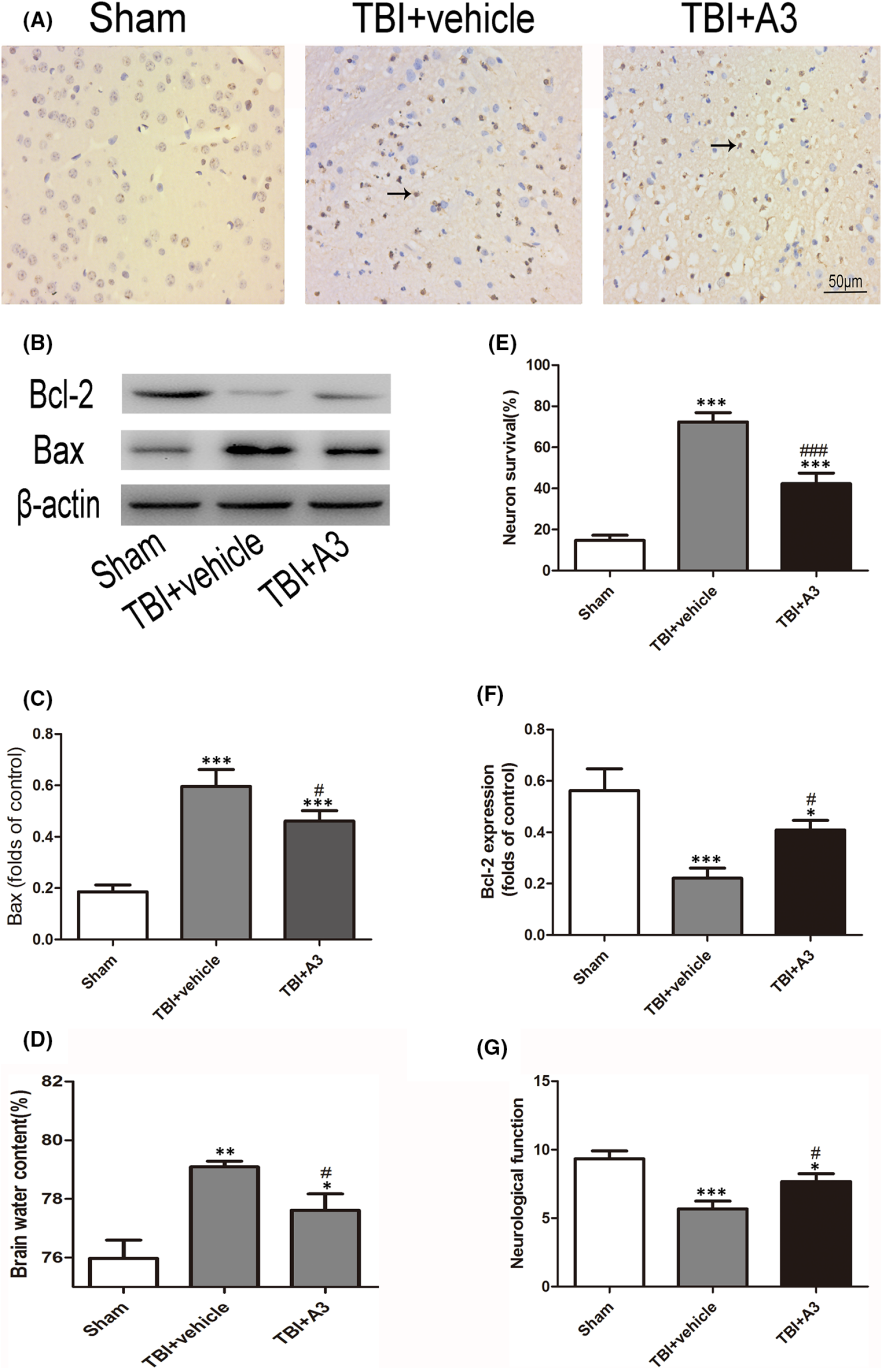
Effects of A3 on apoptosis, brain oedema and neurological function 24 h after TBI. (A, E) Representative photomicrographs of TUNEL staining in the experimental groups. (B, C, F) A3 treatment significantly decreased the Bax protein levels but increased the Bcl‐2 protein levels compared with those in the TBI + vehicle group. (D, G) Impaired brain oedema and neurological behaviour decreased significantly after A3 administration, compared with those in the TBI + vehicle group. Data represent mean ± SD, *p < 0.05, **p < 0.01, ***p < 0.001 versus Sham group; ^#^ *p* < 0.05, ^##^ *p* < 0.01 versus TBI + vehicle group
